# Comprehensive analysis of circRNA expression profile and circRNA-miRNA-mRNA network susceptibility to very early-onset schizophrenia

**DOI:** 10.1038/s41537-023-00399-0

**Published:** 2023-10-10

**Authors:** Huanhuan Huang, Jie Luo, Yanjie Qi, Yuanzhen Wu, Junhui Qi, Xiuping Yan, Gaoyang Xu, Fan He, Yi Zheng

**Affiliations:** National Clinical Research Center for Mental Disorders, Beijing Key Laboratory of Mental Disorders, Beijing Anding Hospital, Beijing Institute for Brain Disorders Capital Medical University, Beijing, People’s Republic of China

**Keywords:** Developmental biology, Biomarkers

## Abstract

To explore the potential role of circular RNAs (circRNAs) in children developing very early-onset schizophrenia (VEOS). Total RNA was extracted from the plasma samples of 10 VEOS patients and eight healthy controls. Expression profiles of circRNAs, micro RNAs (miRNAs), and messenger RNAs (mRNAs) were analyzed using RNA-seq. The interaction networks between miRNAs and targets were predicted using the miRanda tool. A differentially expressed circRNA-miRNA-mRNA (ceRNA) network was further constructed. Gene ontology (GO) and Kyoto Encyclopedia of Genes and Genomes (KEGG) pathway analyses of the target mRNAs in the ceRNA network were performed to predict the potential functions of their host genes. The patient group and the control group were also compared on the regulatory patterns of circRNAs on mRNAs. 1934 circRNAs were identified from the samples and reported for the first time in schizophrenia. The circRNA expression levels were lower in the VEOS group than in the healthy control group, and 1889 circRNAs were expressed only in the control group. Differential expression analysis (i.e., log_2_fold change > 1.5, *p* 0.05) identified 235 circRNAs (1 up-regulated, 234 down-regulated), 11 miRNAs (7 up-regulated, 4 down-regulated), and 2,308 mRNAs (1906 up-regulated, 402 down-regulated) respectively. In VEOS, a ceRNA network with 10 down-regulated circRNA targets, 6 up-regulated miRNAs, and 47 down-regulated mRNAs was constructed. The target genes were involved in the membrane, the signal transduction, and the cytoskeleton and transport pathways. Finally, different expression correlation patterns of circRNA and mRNA in the network were observed between the patient group and the control group. The current research is the first to reveal the differentially expressed circRNAs in the plasma of VEOS patients. A circRNA-miRNA-mRNA network was also conducted in this study. It may be implied that the circRNAs in this network are potential diagnostic biomarkers for VEOS and they play an important role in the onset and development of VEOS symptoms.

## Introduction

Schizophrenia has been widely understood as a disorder related to the interaction of genetic and environmental factors. Additionally, very early-onset schizophrenia (VEOS) refers to schizophrenia with onset before 13 years of age^1^. Clinically, it is characterized by positive (i.e., delusion, hallucination, disorganized thoughts and catatonic behaviour) and negative symptoms (i.e., loss of normal functioning, such as the loss of speech or facial expressions). The pathogenesis of the disorder remains largely unknown, and its course is usually long-term, with severe and progressive impairments in cognitive processing^2^. As the incidence of mental disability and poor outcomes is also higher in this disorder^3^, early diagnosis and identification is very important^4^. Currently, the diagnosis of VEOS mainly relies on clinicians tracking patients’ medical history and clinical symptoms through interviews and auxiliary examinations. Based on the International Classification of Diseases 11th Revision (ICD-11) and the Diagnostic and Statistical Manual of Mental Disorders (Fifth Edition) (DSM-5), VEOS has been regarded as a childhood type of adult schizophrenia diagnosed by the criteria for adult schizophrenia, focusing on hallucinations, delusions and disorders of thinking^5^.

Despite the comparability of VEOS and adult-onset schizophrenia (AOS) in terms of schizophrenia-specific symptoms^6^, empirical evidence supports that there are differences between schizophrenia in children and adults. Indeed, the heterogeneity of AOS and VEOS has been widely evidenced since the 1980s^7^. Compared to AOS, the effect of VEOS on patients’ cognitive and social development start at an earlier age^3,8,9^. Moreover, more neurotic syndromes, conduct disorders and affective symptoms have been found in children patients compared to adults developing schizophrenia^6^. Notably, VEOS is widely confused with other common developmental disorders such as autism spectrum disorder (ASD) and intellectual disabilities, due to the early onset time and the atypical clinical symptoms demonstrated by the patients,. Hence, based on its insidious characteristics compared to AOS^10^, VEOS may be considered a disorder with frequent cases of missed diagnosis and misdiagnosis. Taken together, it is valid to propose that current difficulties in VEOS diagnosis highlight the urgent need for research on its objective biochemical, physiological and pathological indicators^11^.

Indeed, no study to our knowledge has yet investigated the biomarkers of VEOS due to its low prevalence^12^. This may also be explained by the diagnostic difficulties of VEOS illustrated above, which further emphasizes the value of research on VEOS. More importantly, taken that previous literature has evidenced the effectiveness of early intervention in relieving the symptoms of schizophrenia^13^, it may be implied that improving the accuracy and efficacy of VEOS diagnosis would contribute to the treatment effectiveness of schizophrenia. Hence, despite the current lack of empirical evidence on VEOS in particular, investigations on its biomarkers should be conducted for VEOS.

Taking the physiological approach, evidence has highlighted that approximately 90% of genes in eukaryotic genomes are transcribed genes. However, only 1–2% of these genes encode proteins. By contrast, the majority of the others are transcribed as non-coding RNAs (ncRNA)^14^. Particularly, ncRNAs refers to RNA molecules that are transcribed from the genome and do not encode proteins, and their role in the regulation of human gene expression is of great interest^15^. To exemplify, Circular RNA (circRNA) is a special type of non-coding RNA with stability, specificity and conservation^16^. It acts as an endogenous competing RNA to exert a sponge effect to absorb miRNA and thereby affect mRNA levels, indirectly regulating gene expression^17^. Also, it binds to RNA-binding protein (RBP) to participate in transcriptional regulation. In recent years, vitro transcribed circRNAs have received much attention in research on schizophrenia^18–21^. For example, Zimmerman et al.^18^ found that circHomer1a expression was significantly downregulated in neurons derived from induced pluripotent stem cells of schizophrenia and bipolar disorder patients. Moreover, circHomer1a was also significantly downregulated in the orbitofrontal gyrus and dorsolateral prefrontal cortex (DLPFC) of schizophrenia patients. It binds to the neuronal RBP HuD and affects the expression of synaptic structures in the prefrontal cortex. According to Mahmoudi et al.^19^, multiple circRNAs (i.e., mostly downregulated) were differentially expressed in the DLPFC of schizophrenia patients. Particularly, the host genes of these circRNAs were enriched in pathways related to neurogenesis, dendrite morphology and development, neuronal differentiation and brain development^19^. In addition, many studies have detected differentially expressed circRNAs in the blood and blood cells of schizophrenia patients. Recently, Yao et al.^20^ evidenced hsa_circRNA_104597 to be significantly downregulated in patients with schizophrenia and upregulated after systematic treatment, highlighting its role as a potential diagnostic and therapeutic biomarker for schizophrenia in peripheral blood mononuclear cells (PBMCs). Tan et al.^21^ also detected 44 differentially expressed circulating RNAs in the plasma of patients developing schizophrenia. Therefore, circRNA may become a good biomarker for the diagnosis and treatment of schizophrenia.

Overall, circRNA and its constructed circRNA-miRNA-mRNA regulatory network (ceRNA network) may play an important role in the pathogenesis of schizophrenia. To sepecify, their differential expression in peripheral blood may provide new ideas for the exploration and identification of schizophrenia’s early diagnosis and treatment targets. Concerning current research is still limited, further exploration and verification of previous studies are needed. In addition, the investigation of the younger age group developing schizophrenia may allow a research opportunity of studying the aberrant origin of this disorder. Therefore, in this study with children developing VEOS, we aim to use RNA-seq to analyze the expression profiles of circRNAs, miRNAs and mRNAs, to construct a circRNA-miRNA-mRNA network, to identify the risk genes, and to perform KEGG and GO analysis on target genes in the ceRNA network.

## Materials and methods

### Sample recruitment

Eight inpatients at Beijing Anding Hospital were recruited as the VESO group of the current study. All participants were first-episode schizophrenia patients diagnosed by two attending psychiatrists with a title of the associate chief physician or higher, according to the diagnostic criteria of schizophrenia in DSM-5^22^. Only children aged below 13 were involved. The exclusion criteria were: (a) having severe physical illness; (b) demonstrating a comorbidity with other psychiatric disorders, such as depressive disorders, bipolar disorder, and other developmental disorders (i.e., autism spectrum disorders, intellectual disability); (c) having previously undergone antipsychotic or other medication treatments, and (d) being in other situations considered inappropriate for the inclusion in the study by the researchers. Ten participants who had never been diagnosed for any physiological or psychological disorders were recruited for the health control group through community and school contacts. Ultimately, the study recruited 10 patients with VEOS and 8 healthy controls matched by age and gender. Their demographic characteristics are presented in Table 1. The project was conducted according to the ethical guidelines in the Declaration of Helsinki, and its subsequent amendments were approved by the Ethics Committee of Beijing Anding Hospital (Ethical number: 2016103FS-2). Signed informed consent was acquired from all subjects and their parents before the study.

**Table 1:** Sample characteristics.

	VEOS (*n* = 10)	Control (*n* = 8)
Age, mean (SD)	13 ( ± 2)	12 ( ± 1.7)
Male, *n* (%)	4 (40)	5 (62.5)
Female, *n* (%)	6 (60%)	3 (37.5)
Age of onset, mean (SD)	12.2 ( ± 1.3)	N/A
PANSS Total Score, mean (SD)	84.3 ( ± 21.8)	N/A
PANSS positive score, mean (SD)	22 ( ± 7.2)	N/A
PANSS negative score, mean (SD)	19.5 ( ± 7.9)	N/A
PSP score, mean (SD)	38.1 ( ± 12.3)	N/A

*VEOS* very early-onset schizophrenia, *PANSS* Positive and Negative Syndrome Scale, *PSP* personal and social performance scale, *N/A* not applicable.

### RNA extraction, library construction and sequencing

Total RNAs were isolated from participants’ peripheral blood using the PAX Blood RNA kit (QIAGEN, Germany) according to the recommended protocol. Ribosomal RNAs (rRNAs) were removed using the Epicentre Ribo-ZeroTM kit, and the integrity of RNA was assessed using the RNA Nano 6000 Assay kit for the Bioanalyzer 2100 system (Agilent Technologies, CA, USA). The purity of the RNAs isolated was further verified with a NanoPhotometer® spectrophotometer (IMPLEN, CA, USA). Pair-end sequencing with 150-bp reads was performed on the Illumina HiSeq 2500 platform. A small RNA library was prepared using NEBNextR Multiplex Small RNA Library Prep Set for Illumina R (NEB, USA) following recommendations from the manufacturer. We used Agilent Bioanalyzer 2100 system to assess the library quality, and subsequently, the library was sequenced on Illumina Hiseq 2500 platform for generating 50-bp single-end reads.

### RNA-seq data analysis

Firstly, we used the FastQC program^23^ to assess the quality of the raw sequencing reads. Secondly, quality control on the reads was processed to remove adaptor sequences. Thirdly, the 5′ and 3′ end low-quality sequences were also trimmed by Trim Galore (http://www.bioinformatics.babraham.ac.uk/projects/trim_galore/). The remaining high-quality clean reads were aligned to the human genome by STAR^24^ with a UCSC version 27 gene annotation file ftp://ftp.ebi.ac.uk/pub/databases/gencode/Gencode_human/release_27/gencode.v27.annotation.gtf.gz. We used RNA-seq by Expectation-Maximization (RSEM)^25^ to calculate the reads count for each gene expressed, and normalized them by transcript per million (TPM). Meanwhile, we used the FastQC program to evaluate the miRNA raw sequencing reads, and employed the Trim Galore software to clean low-quality reads. Then, the miRDeep2 software^26^ with default parameters was used to identify the mature miRNA from the miRBase database (v.22, http://www.mirbase.org)^27^. Lastly, Mapper.pl script was used to map the clean reads to the human genome, and quantifier.pl was used to quantify the number of the miRNA.

### Detection and annotation of circRNAs

We adopted both the CIRCexplorer2 program^28^ and the find circ program^29^ with default settings to identify reliable candidate circRNAs. The total number of back-spliced reads (i.e., supporting particular head-to-tail junctions) was used as an absolute measure of circRNA abundance. CircRNAs expressed with at least two back-spliced reads in more than half of the samples were included for the downstream analysis. We observed a high consistency of common circRNA expressions measured by CIRCexplorer2 and find circ (Supplementary Fig. 1). All circRNAs identified from sequencing data were intersected with known gene annotations (GENECODE V27 for humans), and were classified based on the type of intersection with a known transcript.

### Differential expression analysis

Following the alignment and the filtering of low expressed molecular (i.e., read count < 5 for mRNA, read count < 2 for circRNA, read count < 50 for miRNA), differential expression (DE) tests were performed using the DESeq2 package^30^. Expressions of transcripts with a fold change > 1.5 and a *p*-value < 0.05 was considered statistically significant. Regarding result visualization and reporting, volcano plots were used to identify changes in expression comparison.

### Comparison of circRNA expression between schizophrenias and controls

The distributions of circRNA expressions in the VEOS group and the matched control group were assessed. Specifically, we normalized the original back-spliced junction reads of each circRNA in each sample into transcript per million reads by dividing the total number of mapped reads. The distribution of the average expression value of each circRNA in different groups was plotted and compared, and the differences between the two groups were analyzed by Student’s t-test. Then, we used a log of fold change for each circRNA to indicate the ratio of the expression levels between the two groups. Finally, circRNAs that were exclusively expressed were detected with the same threshold highlighted in the above section.

### Construction of the circRNA-miRNA-mRNA regulatory network

We constructed the miRNA-circRNA network and miRNA-mRNA network based on the DE results. The differentially expressed miRNA-mRNA and miRNA-circRNA networks were predicted with miRanda (version 3.3a)^31^. Specifically, miRanda predicted miRNA targets based on sequence complementarity. The maximum binding free energy of -20 kcal/mol for miRNA targets interaction was used as the threshold for the predictions in miRanda. Although the expression levels of circRNAs, as the sponge of miRNAs, usually do not affect the expression of miRNA, we selected circRNA-miRNA pairs with the constraint of inverse expression relationships as the miRNA-mRNA pairs. Finally, we used Cytoscape (v 3.6.1)^32^ to visualize the regulatory network.

### Gene-sets enrichment analysis

GO and KEGG pathway annotations were conducted by clusterProfile, and R package were adopted for analyzing and visualizing the profiles of genes and gene clusters^33^. Particularly, the functional classification and enrichment of gene clusters were conducted according to hypergeometric distribution. The GO terms and KEGG pathways with a threshold of FDR < 0.05 and p-value < 0.01 were considered significant enrichment.

## Results

### Profiling the circRNA expression in schizophrenic patients and matched controls

**Fig. 1:**
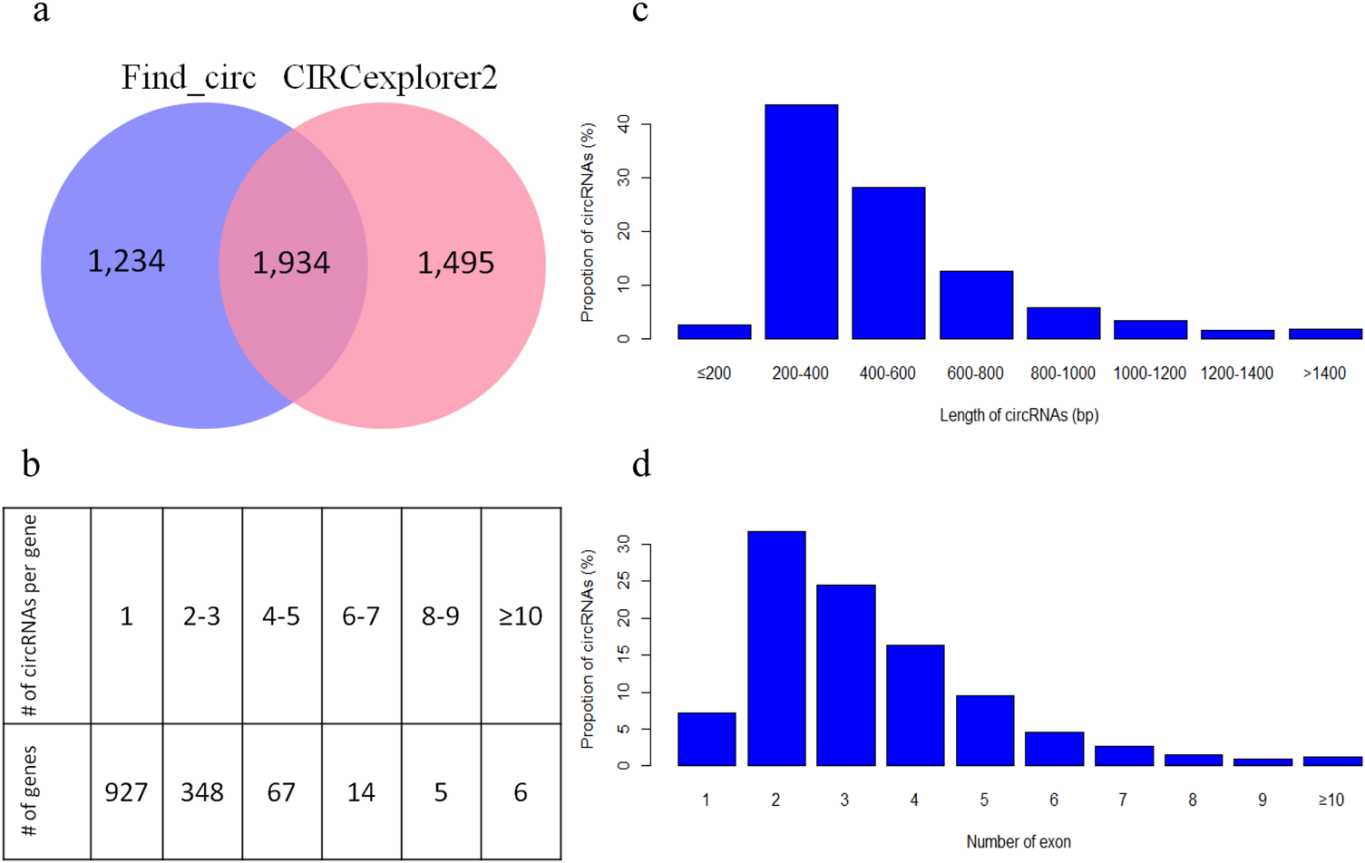
Characterization of circRNAs. **a** Find circ and CIRCexplorer 2 identified 3168 and 3429 circRNAs, respectively, of which 1934 were identified by both algorithms; **b** Six genes can transcribe more than 10 circRNAs; **c** most circRNAs are concentrated between 200 and 800 bp in length; **d** the number of exons contained in circRNAs is concentrated in the range of 2–5.

We used high-throughput sequencing methods to systematically profile circRNA expression in all samples with two well-accepted circRNA identification algorithms (Table 2). In total, 1934 highly expressed circRNAs from the two groups were detected by both find circ and CIRCexplorer2 with a high consistency of expression measurements (Fig. 1a and Supplementary Fig. 1). Among them, we observed 1927 (99.6%) circRNAs annotated into protein coding region. 11 circRNAs intersected with sense intronic region, and 5 circRNAs intersected with lncRNA. As shown in Fig. 1b, most of the genes generated less than 3 circRNA transcripts, whereas 6 genes (i.e., *RF00019, ABCC4, XP01, UBAP2, PICALM*, and *DENND4A*) were found to transcribe more than 10 circRNAs. As shown in Fig. 1c, the lengths of circRNA transcripts were mostly distributed between 200 and 400 bp. In addition, the number of exons in circular transcripts were ranged mainly from 2 to 5 (Fig. 1d).

**Table 2:** circRNA identifications in each sample by two programs.

Sample ID	Find_circ	CIRCexplorer2	Overlap
P04	4514	5915	1979
C09	3191	5973	1674
P07	9507	8323	3951
P10	3448	3880	1275
C06	9106	8657	3824
P08	1657	3479	701
C02	8037	8480	3425
C05	4179	3980	1533
P06	3927	7226	2144
C01	4000	5983	1861
P03	6524	5827	2563
C07	8902	8978	3789
C03	7563	7598	3189
P09	3281	3631	1198
C04	7026	5799	2528
P02	5580	5707	2231
P01	5376	4824	1993
P05	3255	3789	1223

### Global circRNA levels dramatically decrease in schizophrenic patients compared to controls

**Fig. 2:**
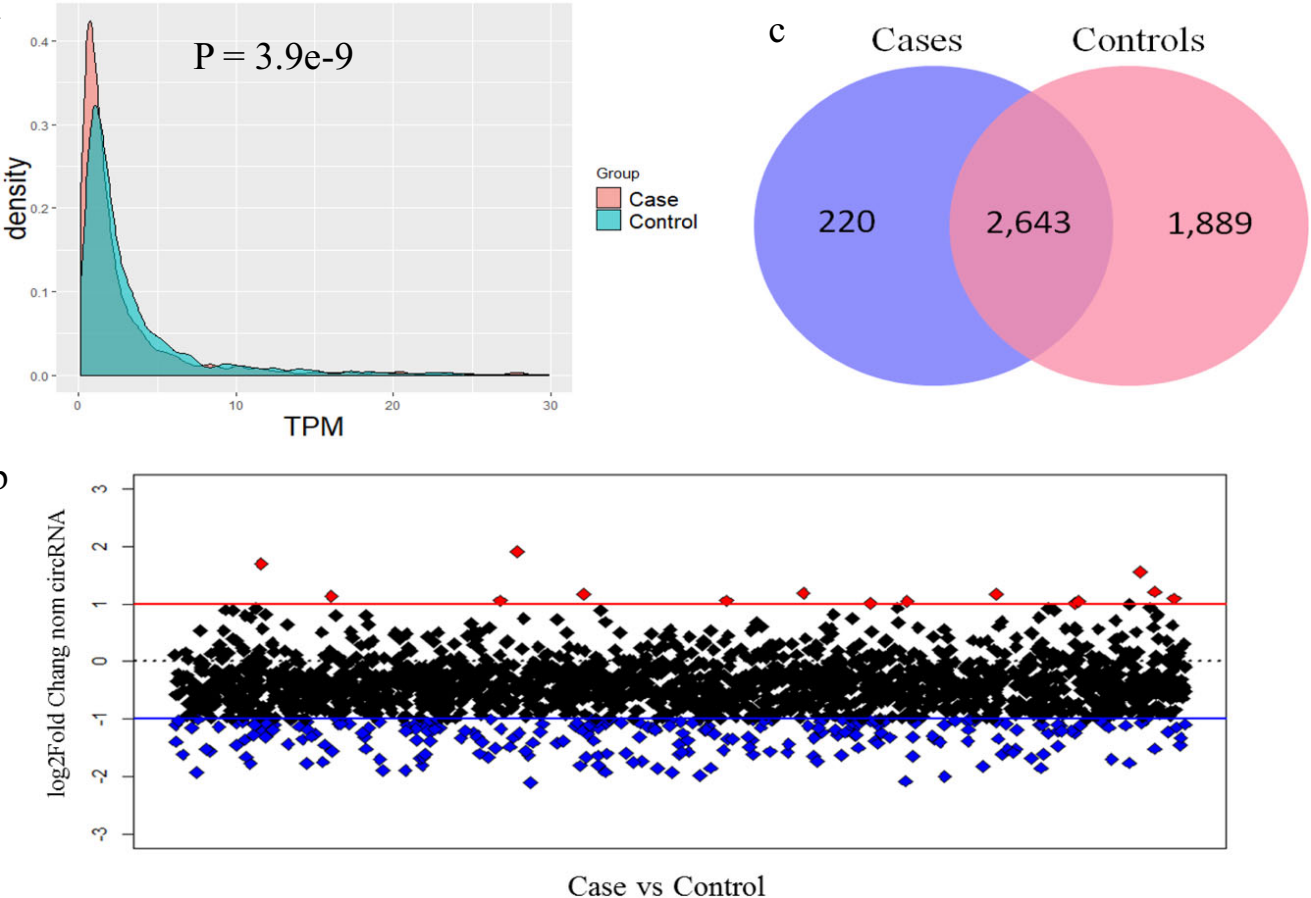
Comparison of circRNA expression between groups. **a** There is a trend of low expression of circRNAs in schizophrenic patients; **b** Using |fold change |> 2 as the threshold, red dots indicate circRNAs expressed up - regulated in cases and blue dots indicate circRNAs expressed down - regulated in cases; **c** Fewer circRNAs were produced in the case group than in the control group, with 1889 circRNAs appearing only in the control group.

To systematically quantify and evaluate the abundance of circRNAs in the schizophrenia and the control groups, we normalized circRNA back-spliced reads into Transcripts Per Million reads (TPM) (see Methods). The density curves for average circRNA TPM values in the two groups were compared, and the global expression profiles in the VEOS group were found to be significantly lower than that in the control group (Fig. 2a). To further investigate the trends in circRNA expression levels, we compared the plotted circRNA log_2_ fold changes between the VEOS group and the control group (Fig. 2b). We next quantified circRNAs that exclusively expressed in one group using the same criteria. As shown in Figs. 2c, 220 and 1,889 circRNAs were exclusively detected in the VEOS group and the control group, respectively.

### Identification of differentially expressed RNAs in schizophrenias

**Fig. 3:**
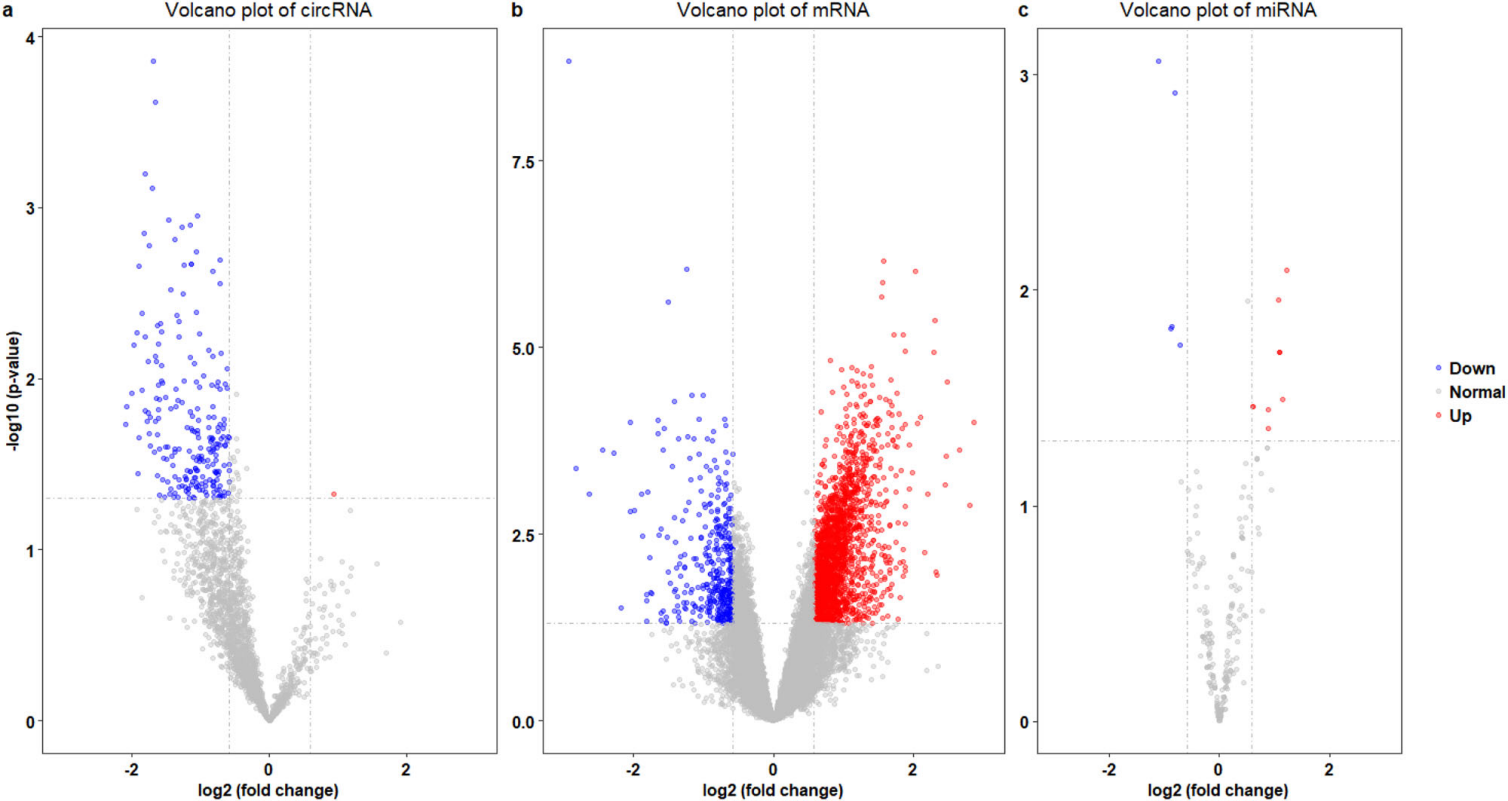
Differential expression analysis of circRNA, mRNA, miRNA. **a** Volcano plot of circRNA; **b** Volcano plot of mRNA; **c** Volcano plot of miRNA; Red indicates upward adjustment, blue indicates downward adjustment.

RNA-seq data collected from the participants allowed further analyses on miRNA, circRNA and mRNA expression profiles. DE analyses were conducted by DEseq2 with normalization and model fitting. Subsequently, Wald-tests were used to evaluate the statistical significance of the differentially expressed RNAs. RNAs with a fold change > 1.5 and a *p*-value < 0.05 were considered as significantly differentially expressed. Comparing the VEOS and the control groups, we identified 2308 aberrantly expressed mRNAs (i.e., 1906 upregulated, 402 downregulated). Meanwhile, 1 upregulated and 234 downregulated circRNAs were found to be differentially expressed. Additionally, 11 aberrantly expressed miRNAs (i.e., 7 upregulated and 4 downregulated) were also detected. We used volcano plot to visualize the RNA expression variability between the two groups and the statistical significance (Fig. 3). Among the genes related to VEOS, we evidenced *IGF1R* as a host gene of a deficiently expressed circRNA (hsa_circ_0005035, log_2_ FC = -0.81, *P* = 0.02).

### Establishment of the DE circRNA-miRNA interaction networks

To better understand the regulatory effects of aberrantly expressed circRNA, we identified the putative circRNA-miRNA interaction networks. The searching for microRNA response elements (MREs) was based on differentially expressed circRNAs and miRNAs. Only interaction networks with a maximum binding free energy of -20 kcal/mol according to the MiRanda^31^ predictions were reported. A total of 24 interaction networks were detected, including 10 miRNAs and 16 circRNAs. 58.3% of these networks showed opposite expression patterns (i.e., one was upregulated as the other was downregulated, Table 3). In detail, we found 6 upregulated miRNAs targeting 10 downregulated circRNAs. The most concentrated connections were observed between has-miR-16-5p and four circRNAs (i.e., hsa_circ_0000638, hsa_circ_0005035, hsa_circ_0001200, and hsa_circ_0001789).

**Table 3:** miRNA target sets predictions.

miRNA	miRNA status	circRNA	circRNA status	Total Energy	Positions
hsa-let-7a-5p	Up	chr17_67945408_67975958	Up	−22.32	1049
hsa-let-7a-5p	Up	chr2_159729008_159752571	Down	−26.91	297
hsa-let-7b-5p	Up	chr17_67945408_67975958	Up	−20.39	1047
hsa-let-7b-5p	Up	chr1_150230570_150231926	Down	−20.57	204
hsa-let-7b-5p	Up	chr2_159729008_159752571	Down	−24.8	297
hsa-miR-16-5p	Up	chr17_67945408_67975958	Up	−21.93	972
hsa-miR-16-5p	Up	chr15_76274411_76295737	Down	−21.2	693
hsa-miR-16-5p	Up	chr15_98707561_98708107	Down	−20.88	146
hsa-miR-16-5p	Up	chr21_44855209_44861271	Down	−20.24	168
hsa-miR-16-5p	Up	chr8_37877108_37877551	Down	−20.92	354
hsa-miR-18a-5p	Up	chr8_99502835_99511512	Down	−21.56	243
hsa-miR-18a-5p	Up	chr3_15411244_15415942	Down	−23.66	129
hsa-miR-18a-5p	Up	chr6_147260614_147278204	Down	−24.6	324
hsa-miR-92a-3p	Down	chr14_31127784_31133675	Down	−23.03	836
hsa-miR-92a-3p	Down	chr4_55411613_55417985	Down	−20.76	176
hsa-let-7g-5p	Up	chr14_31127784_31133675	Down	−20.6	88
hsa-let-7g-5p	Up	chr2_159729008_159752571	Down	−31.76	297
hsa-let-7i-5p	Up	chr14_31127784_31133675	Down	−22.24	86
hsa-let-7i-5p	Up	chr2_159729008_159752571	Down	−26.91	297
hsa-miR-30b-5p	Down	chr5_109713519_109729513	Down	−21.36	251
hsa-miR-654-3p	Down	chr10_26500818_26503274	Down	−21.12	45
hsa-miR-654-3p	Down	chr5_55645788_55664863	Down	−20.32	28
hsa-miR-1271-5p	Down	chr5_154029488_154034967	Down	−21.49	104
hsa-miR-1271-5p	Down	chr3_15411244_15415942	Down	−20.01	397

*Note:* The inversely expressed circRNA-miRNA pairs were shown in italic.

### Construction of the differentially expressed circRNA-miRNA-mRNA network

**Fig. 4:**
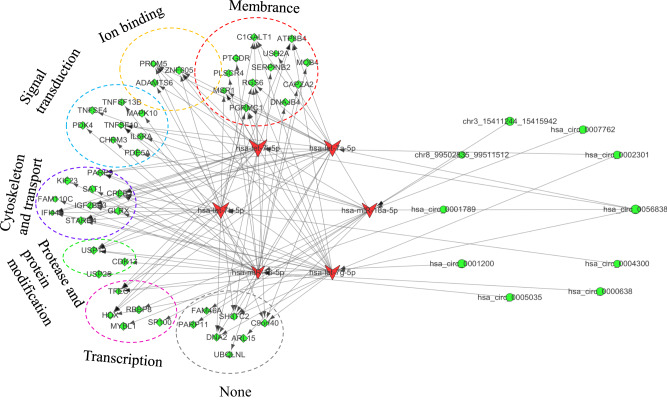
Differential expression with specific direction circRNA-miRNA-mRNA triple regulatory network.

The identification of dysregulated RNAs contributed to the exploration of the potential regulatory functions of the ceRNA network in schizophrenia. To investigate the mechanism of ceRNA regulation, we first identified the differentially expressed miRNA-circRNA and the differentially expressed miRNA-mRNA interaction networks respectively. After, miRNAs connected to both circRNAs and mRNAs were identified, allowing the construction of a differentially expressed circRNA-miRNA-mRNA network. Based on miRanda with a maximum binding free energy of -20 kcal/mol, we identified 11 differentially expressed miRNAs and 6,368 mRNAs. All differentially expressed circRNA-miRNA-mRNA interaction networks we obtained are presented in Supplementary Fig. 2. As circRANs competitively adsorb miRNAs, we considered that circRNAs and miRNAs would have opposite regulatory functions. Particularly, the differentially upregulated circ RNAs would interact with the differentially downregulated miRNAs, and vice versa. Similarly, the regulation network between miRNAs and mRNAs would follow the same pattern. This left 10 circRNAs, 6 miRNAs and 47 mRNAs with inverse expression relationships in the network (Fig. 4). Furthermore, for the schizophrenia-related ceRNA network, we performed functional annotation for the downstream genes that were regulated by circRNAs. The top overrepresented terms included the membrane, the cytoskeleton and the transport and signal transduction (Fig. 4).

### The disturbed circRNA-mRNA correlation pattern in schizophrenia

**Fig. 5:**
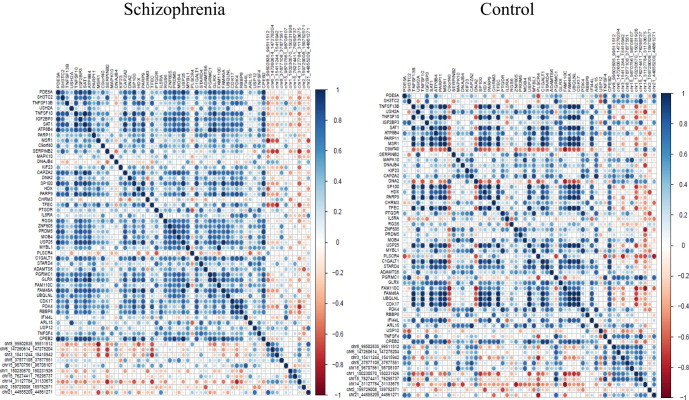
Heat map of molecular regulatory correlations between case control groups.

**Fig. 6:**
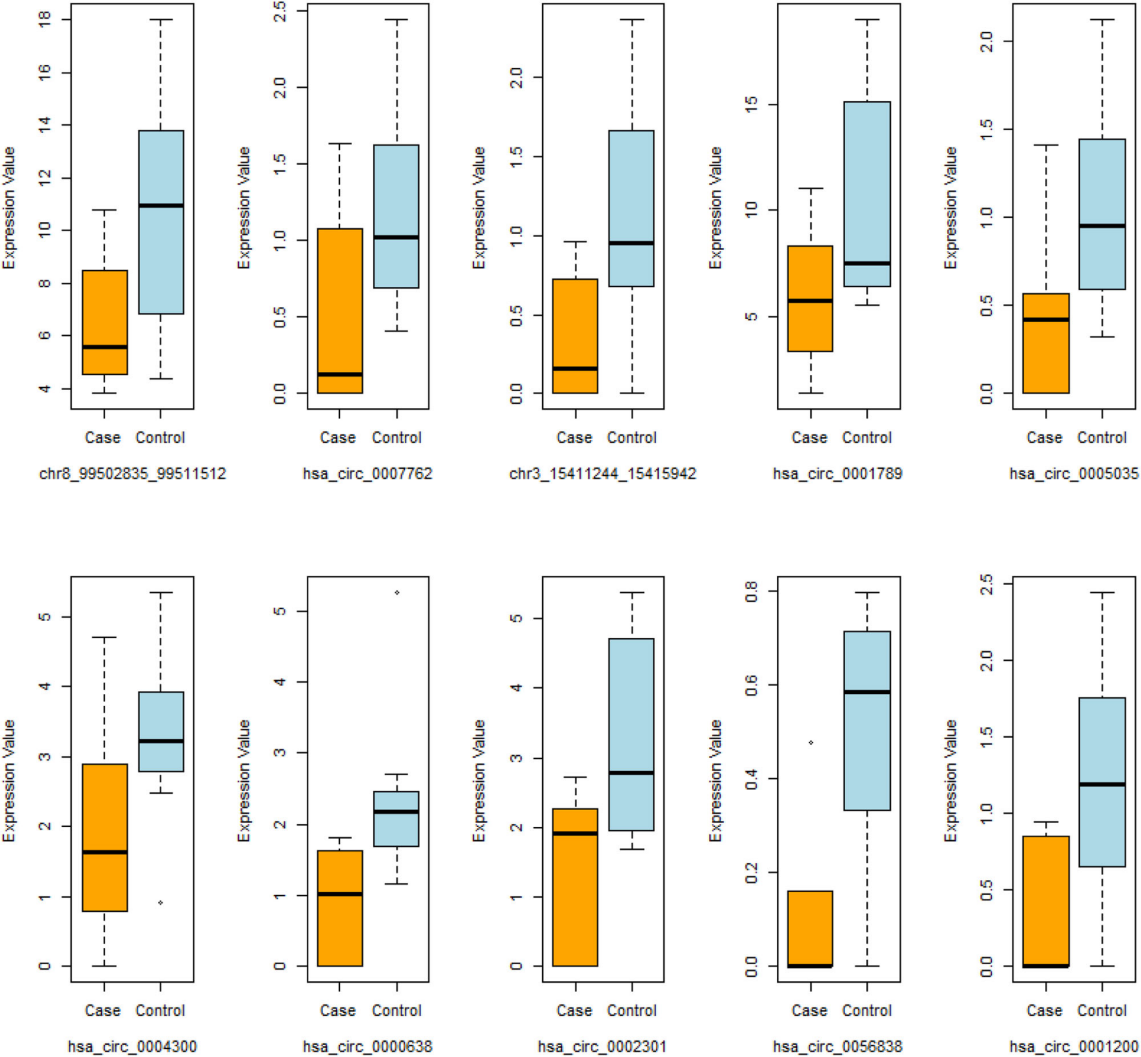
Candidate circRNA box plot.

Finally, we investigated the expression correlation pattern of circRNA-mediated ceRNA network in the VEOS group and the control group respectively. The pair-wise correlation coefficients between the 10 circRNAs and the 47 mRNAs were compared and the findings were presented in the heatmap (Fig. 5). Multiple parallel positive correlations in the VEOS group were observed, whereas a chaotic pattern were found in the control group, indicating an interaction between the circRNAs and VEOS symptoms. The distributions of expression level for 10 regulatory circRNAs in schizophrenias and controls were displayed in Fig. 6.

## Discussion

CircRNA is a unique class of endogenous non-coding RNAs generated by non-canonical selective splicing. Thousands of endogenous circRNAs have been identified in humans’ eukaryotic cells^34^. In this study, 1934 circRNAs were identified in the plasma samples of patients with VEOS for the first time. Compared to the healthy controls, the plasma circRNA levels decreased significantly in VEOS patients, and most of the differentially expressed RNAs were downregulated. Among the differentially expressed RNAs, the host genes of circRNAs related to schizophrenia were insulin-like growth factor receptor 1 (IGF1R). Specifically, IGF1R is an important cell surface receptor associated with multiple nervous system functions such as neurodevelopment, neuroprotection and neuronal signal transduction^35^. Previous studies have not only highlighted IGF-1R depletion as a non-neurotransmitter pro-schizophrenic cue in mice^36^, but also confirmed the reduced IGF1R mRNA expression levels in the subependymal zone of schizophrenia patients, with emphasis on its involvement in neurogenesis and pathogenesis^37^. In addition, single nucleotide variants and indels were present in the IGF1R gene in schizophrenic patients^38^, and increased antibody reactivity were found IGF1R in patients’ sera, with an evidenced positive correlation between reactivity and PANSS scores^39^. This study also found that IGF1R expression levels were positively correlated with neurodevelopmental indicators such as neurogenesis, neuronal maturation and synapse formation, whereas the exact role of circRNA in early-onset schizophrenia still needs further investigations.

In addition, circRNAs have been found to act as miRNA sponges to regulate gene expression^40^. Our study added to current findings by identifying differentially expressed circRNAs and their corresponding miRNA binding sites. Particularly, we marked 6 upregulated miRNAs targeting 10 downregulated circRNAs. The most concentrated links were observed between has-miR-16-5p and four circRNAs (i.e., hsa_circ_0000638, hsa_circ_0005035, hsa_circ_0001200 and hsa_circ_0001789). Our finding corresponds to previous research that the precursor form of this miRNA (i.e., miR-16) demonstrates increased expression levels in the superior temporal gyrus (STG) and dorsolateral prefrontal cortex (DLPFC) of patients with schizophrenia^41^. Three unique circRNAs (i.e., hsa_circ_0007763, chr3_15411244_15415942, chr8_99502835_99511512) connected to has-miR-18a-5p were also found to show significant upregulation in schizophrenia, extending the external validity of previous studies^42^.

Due to the competitive adsorption relationship between circRNA and miRNA, we hypothesized that circRNA and miRNA have opposite regulatory roles. Specifically, differentially upregulated circRNA would act on differentially downregulated miRNA, and vice versa. Similarly, the regulatory method applies to miRNA and mRNA. Therefore, we constructed a ceRNA network only for those with inverse regulatory relationships, and we functionally annotated the downstream genes regulated by circRNAs. Existing hypotheses on the etiology of schizophrenia include the classical dopamine transmission disorder hypothesis, the excitation/inhibition balance disorder hypothesis, the glutamatergic N-methyl-D-aspartate receptor dysfunction hypothesis, the immune dysfunction hypothesis, etc.^43^. However, none of these hypotheses fully explains the symptoms of schizophrenia. Our study added to the current understanding of the disorder by highlighting the locationsof the circRNA-regulated genes. Particularly, pathways found by our research include: the membrane, the signal transction and the cytoskeleton and transport pathways.

To start with, the membrane signaling pathways include G protein-coupled receptors (GPCRs) and tyrosine kinase receptors. These receptors transduce signals and affect cell function and metabolism by binding to extracellular molecules or signaling molecules^44^. Many studies have found that dopamine activates membrane signaling pathways by binding to D2 receptors, thereby leading to abnormal excitation and signal transduction of neurons, and subsequently the onset of schizophrenia^45^. The cell membrane pathway in this study contains key genes such as RGS6, PGRMC1 and PLSCR4. Particularly, RGS6 and PGRMC1 are the targets of miRNA let-7g-5p. RGS6 is a member of G protein signaling regulating proteins and is considered a therapeutic target for central nervous system diseases^46^. The PGRMC1-related pathway has been found to associate with cognitive impairment in Alzheimer’s disease, but its role in psychiatric disorders needs further verification^47^. Moreover, the PLSCR4 gene, as a target of miRNA 16-5p, has been verified to associate with schizophrenia in previous studies^48^.

Secondly, the signal transduction pathway includes multiple intracellular signal transduction pathways, such as the cAMP-PKA-CREB signaling pathway and the PI3K-Akt-mTOR signaling pathway. These signaling pathways regulate gene expression and metabolism of cells, and affect cell function and response. Studies have found that abnormal expression of the PI3K-Akt-mTOR signaling pathway is associated with the pathogenesis of schizophrenia, and inhibitors of this pathway may have therapeutic effects^49^. The signal transduction-related key genes in this study include TNFSF10, CHRM3, MAPK10 and TNFSF13B. Gene expression microarray results suggested that TNFSF10, a gene associated with p53-mediated cell apoptosis, is a candidate gene for schizophrenia^50^. Furthermore, it was reported that a single nucleotide polymorphism near CHRM3 is associated with functional connectivity abnormalities in treatment-refractory schizophrenia patients^51^. In addition, while TNFSF13B was found to be upregulated in the cerebral cortex and the hippocampus of female mice with schizophrenia-related symptoms^52^, MAPK10 located on chromosome 4 has also been evidenced to associate with psychiatric disorders in previous studies^53^.

Lastly, the cytoskeleton and transport pathways play an important role in the normal function and morphological maintenance of neurons. Existing studies have suggested a reduction in the stability of microtubules in the brain tissue of schizophrenia patients, which leads to the abnormal morphology and functioning of the neurons, thereby affecting the normal signal transduction and synaptic plasticity of neurons^54^. In addition, other research has also shown that antipsychotic drugs regulate the normal functions and morphology of neurons by affecting the functions of the cytoskeleton and transport pathways, and subsequently relieving the symptoms of schizophrenia patients^55^. Genes related to the cytoskeleton and transport pathway include IFI44L, SAT1, etc. IFI44L gene is a target of miRNA let-7g-5p. According to current findings from genome-wide analysis, IFI44L may be considered as a risk gene for schizophrenia^56^. In addition, SAT1 gene is a target of miRNA 16-5p, with previous studies evidencing it as a biomarker of suicidal behavior. To elaborate, SAT1 expression was found to decrease in patients with depression, and it induces iron death of astrocytes associated with Alzheimer’s disease^57^, while playing an important role in neuropsychiatric diseases^58^.

Taken together, to further explore whether the regulatory mode of circRNA on mRNA in the above network differed between the VEOS group and the healthy control group, we studied the expression correlation between circRNA and mRNA in the two groups respectively. Differences between the two groups were observed. This suggests the influence of VEOS on molecular expression regulation, and further confirms that the 10 candidate circRNAs identified in this study were significantly differentially expressed between the VEOS group and the control group (Fig. 6). Based on existing research, the host gene of hsa_circ_0005035 (i.e., IGF1R) has been observed to play an important role in the pathogenesis of schizophrenia, and STXBP5 (i.e., the host gene of hsa_circ_0007762) were involved in the docking and fusion of synaptic vesicles and presynaptic membranes. Meanwhile, alterations in STXBP5 were found to be associated with ASD and other neurodevelopmental and neuropsychiatric disorders^59^. The host gene of chr8_99502835_99511512 is VPS13B, and VPS13B deletion appears related to schizophrenia^60^. The signaling pathways and key genes found above may play an important role in the early onset of childhood schizophrenia and guide further research on the underlying mechanism of VEOS.

One major limitation should be highlighted in the current research. Specifically, the sample size of our study was relatively small. This was due to the lack of previous research in the field, as well as the high cost of high-throughput sequencing. Accordingly, it is recommended that further verification on the circRNAs and the ceRNA networks observed in the current study should be conducted with more participants developing VEOS. Despite the advances made by our research in understanding the physical characteristics of VEOS, this field remains largely unexplored. Subsequently, we would give our top priority to future investigations on verifying the expression of the 10 candidate circRNA, the circRNA-microRNA binding, and the circRNAs-microRNAs colocalization assay. RT-qPCR, circRNA-specific probe RNA in vivo precipitation and Fluorescence In Situ Hybridization (FISH) would be adopted for these investigations respectively. Corresponding to the ultimate goal of preventing VEOS occurrence and improving its prognosis, in addition to finding more physiological characteristics of VEOS, specific attention should also be paid to the clinical application in terms of VEOS diagnosis and treatments.

Overall, it should be emphasized that the accurate regulation of gene expression is essential for maintaining balanced and normal physiological activity. Notably, this study not only provides insights into the novel targets for the diagnosis of VEOS, but also contributes to the pathological physiology restoration by the construction of the circRNA-miRNA-mRNA networks. Meanwhile, the proposal of ceRNA and RNA binding networks further enhances current understanding of the transcriptome and RNA regulatory networks, underlining a new perspective for exploring the occurrence and development of various diseases.

## Conclusion

In conclusion, our study provided the first empirical evidence of the differentially expressed circRNAs with the plasma of VEOS patients, and we constructed a circRNA-miRNA-mRNA network for VEOS. The findings of our research highlight that circRNAs in this network may be potential diagnostic biomarkers and may play an important role in the onset and development of VEOS.

## Availability of data and materials

The data that support the findings of this study are available on request from the corresponding author. The data are not publicly available due to privacy or ethical restrictions.

### Supplementary information

Supplemental Tables

Supplemental Figure 1

Supplemental Figure 2
