# Supplementary material for: Comprehensive analysis of circRNA expression profile and circRNA-miRNA-mRNA network susceptibility to very early-onset schizophrenia
Source: Schizophrenia (Heidelb). 2023 Oct 10;9(1):70. doi: 10.1038/s41537-023-00399-0 (PMC10564922; doi:10.1038/s41537-023-00399-0)
Supplement: Supplementary file 1 — Supplemental Tables [file 41537_2023_399_MOESM1_ESM.docx]

**Supplementary table 1: Sequencing quality statistics**

| Filename | Raw_reads | Clean_reads | Raw_Q20 | Raw_Q30 | Clean_Q20 | Clean_Q30 | %GC |
| --- | --- | --- | --- | --- | --- | --- | --- |
| C01_R1 | 45007317 | 44986897 | 99.99% | 98.61% | 100.00% | 98.73% | 46 |
| C01_R2 | 45007317 | 44986897 | 99.78% | 96.81% | 99.88% | 97.09% | 46 |
| C02_R1 | 47900628 | 47840329 | 100.00% | 98.19% | 100.00% | 98.35% | 46 |
| C02_R2 | 47900628 | 47840329 | 99.56% | 94.67% | 99.78% | 95.10% | 46 |
| C03_R1 | 54278572 | 54208380 | 100.00% | 98.38% | 100.00% | 98.50% | 50 |
| C03_R2 | 54278572 | 54208380 | 99.51% | 94.47% | 99.76% | 94.88% | 50 |
| C04_R1 | 56838457 | 56771379 | 100.00% | 98.17% | 100.00% | 98.33% | 43 |
| C04_R2 | 56838457 | 56771379 | 99.57% | 95.09% | 99.80% | 95.54% | 43 |
| C05_R1 | 51402660 | 51303961 | 99.99% | 98.05% | 100.00% | 98.22% | 44 |
| C05_R2 | 51402660 | 51303961 | 99.42% | 94.39% | 99.74% | 94.93% | 44 |
| C06_R1 | 54957411 | 54911432 | 100.00% | 98.29% | 100.00% | 98.42% | 50 |
| C06_R2 | 54957411 | 54911432 | 99.60% | 94.73% | 99.79% | 95.12% | 50 |
| C07_R1 | 52956658 | 52888904 | 100.00% | 98.21% | 100.00% | 98.37% | 49 |
| C07_R2 | 52956658 | 52888904 | 99.56% | 94.76% | 99.78% | 95.18% | 49 |
| C09_R1 | 45437348 | 45362544 | 99.99% | 97.90% | 100.00% | 98.11% | 45 |
| C09_R2 | 45437348 | 45362544 | 99.41% | 93.23% | 99.69% | 93.74% | 45 |
| P01_R1 | 64425057 | 64367735 | 99.99% | 97.30% | 100.00% | 97.41% | 48 |
| P01_R2 | 64425057 | 64367735 | 99.88% | 96.46% | 99.94% | 96.82% | 48 |
| P02_R1 | 38441822 | 38409863 | 100.00% | 97.61% | 100.00% | 97.71% | 47 |
| P02_R2 | 38441822 | 38409863 | 99.87% | 96.16% | 99.93% | 96.56% | 47 |
| P03_R1 | 43325416 | 43294821 | 100.00% | 97.31% | 100.00% | 97.40% | 46 |
| P03_R2 | 43325416 | 43294821 | 99.87% | 95.75% | 99.93% | 96.11% | 46 |
| P04_R1 | 58216813 | 58197629 | 99.99% | 98.62% | 100.00% | 98.75% | 46 |
| P04_R2 | 58216813 | 58197629 | 99.79% | 96.54% | 99.89% | 96.87% | 46 |
| P05_R1 | 52857269 | 52820720 | 99.99% | 98.56% | 100.00% | 98.74% | 48 |
| P05_R2 | 52857269 | 52820720 | 99.79% | 96.75% | 99.89% | 97.16% | 48 |
| P06_R1 | 55113429 | 55076590 | 99.99% | 98.57% | 100.00% | 98.68% | 47 |
| P06_R2 | 55113429 | 55076590 | 99.70% | 96.17% | 99.83% | 96.46% | 47 |
| P07_R1 | 67714272 | 67697234 | 100.00% | 97.51% | 100.00% | 97.60% | 48 |
| P07_R2 | 67714272 | 67697234 | 99.92% | 96.70% | 99.95% | 96.96% | 48 |
| P08_R1 | 61303609 | 61262706 | 100.00% | 98.44% | 100.00% | 98.61% | 53 |
| P08_R2 | 61303609 | 61273696 | 99.84% | 97.28% | 99.92% | 97.61% | 53 |
| P09_R1 | 53025540 | 52997879 | 99.99% | 98.42% | 100.00% | 98.59% | 50 |
| P09_R2 | 53025540 | 52997879 | 99.82% | 97.19% | 99.91% | 97.54% | 50 |
| P10_R1 | 72158095 | 72132512 | 99.99% | 98.54% | 100.00% | 98.70% | 45 |
| P10_R2 | 72158095 | 72132512 | 99.83% | 97.19% | 99.92% | 97.52% | 45 |

**Supplementary table 2: Alignment information for each sample**

| **Sample ID** | **Total Input Reads** | **Uniquely mapped reads** | **Uniquely mapped rate** | **Total spliced read** | **circular spliced reads** |
| --- | --- | --- | --- | --- | --- |
| C01 | 44986897 | 40246071 | 89.46% | 21233988 | 104703 |
| C02 | 47840329 | 41654456 | 87.07% | 21947234 | 143343 |
| C03 | 54208380 | 46767771 | 86.27% | 37981214 | 123620 |
| C04 | 56771379 | 51498096 | 90.71% | 19250975 | 102409 |
| C05 | 51303961 | 47644962 | 92.87% | 18119684 | 88721 |
| C06 | 54911432 | 45454044 | 82.78% | 37078709 | 148114 |
| C07 | 52888904 | 45174664 | 85.41% | 36480248 | 161687 |
| C09 | 45362544 | 38155319 | 84.11% | 16342115 | 110730 |
| P01 | 64367735 | 55905369 | 86.85% | 26833222 | 93523 |
| P02 | 38409863 | 33357465 | 86.85% | 21449905 | 101001 |
| P03 | 43294821 | 38142485 | 88.10% | 18033169 | 101094 |
| P04 | 58197629 | 51638257 | 88.73% | 23462804 | 107244 |
| P05 | 52820720 | 47323071 | 89.59% | 24917107 | 76265 |
| P06 | 55076590 | 50070574 | 90.91% | 35770551 | 136028 |
| P07 | 67697234 | 58444685 | 86.33% | 38873258 | 147887 |
| P08 | 61273696 | 29987466 | 48.94% | 18620621 | 58916 |
| P09 | 52997879 | 34147496 | 64.43% | 17436294 | 64797 |
| P10 | 72132512 | 66213905 | 91.79% | 33728699 | 88159 |
