# Supplementary figures and images for: Comprehensive analysis of circRNA expression profile and circRNA-miRNA-mRNA network susceptibility to very early-onset schizophrenia
Source: Schizophrenia (Heidelb). 2023 Oct 10;9(1):70. doi: 10.1038/s41537-023-00399-0 (PMC10564922; doi:10.1038/s41537-023-00399-0)

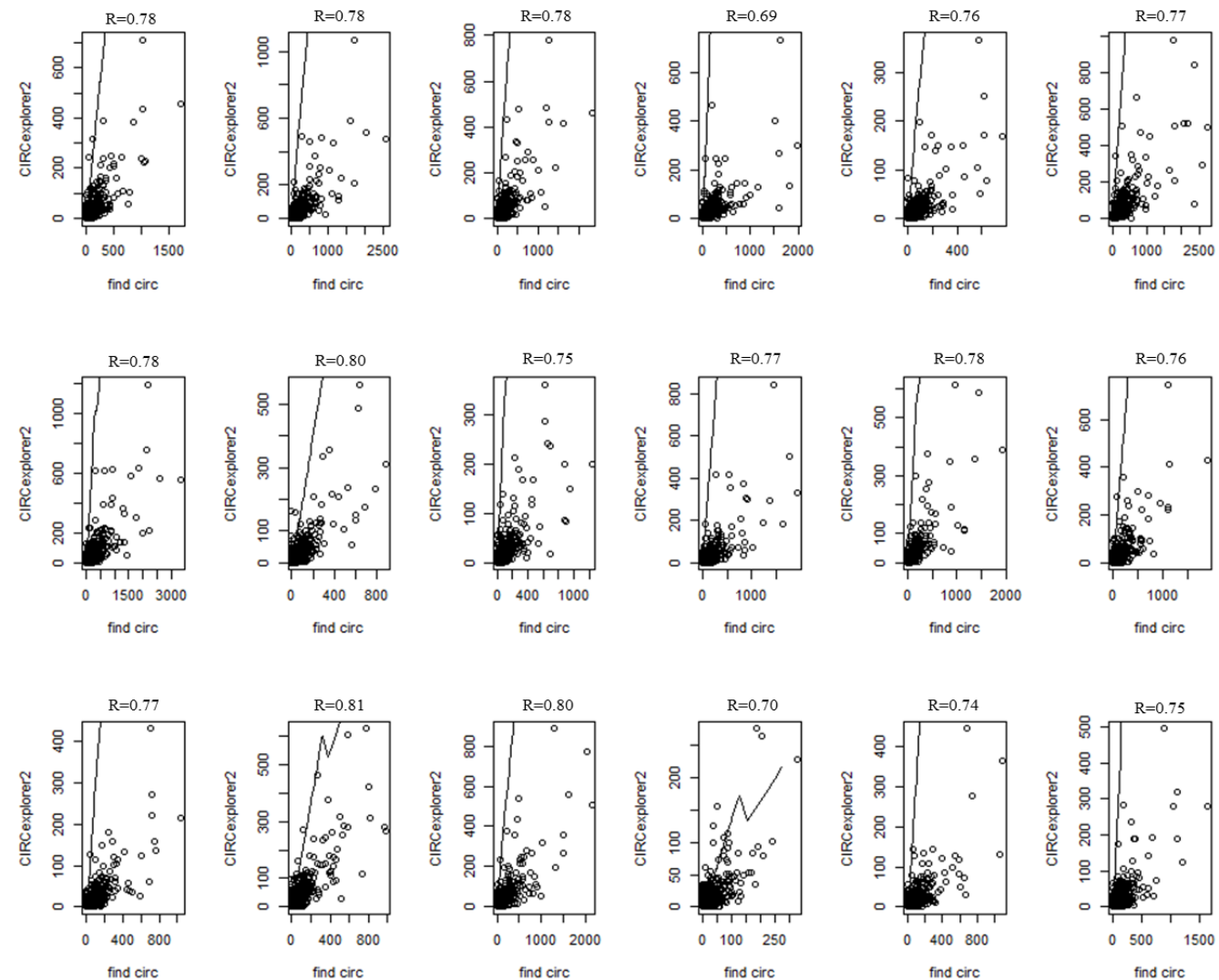

Supplemental Figure 1 Reproducibility of cyclic RNA identification techniques

Supplement: Supplementary file 2 — Supplemental Figure 1 [file 41537_2023_399_MOESM2_ESM.pdf]
